# Oleuropein Modulates ESR2/ERβ-Associated microRNA Responses and Alters 5-Fluorouracil Activity in the Ehrlich Ascites Tumor Model: An In Vivo and Ensemble Docking Study

**DOI:** 10.3390/biom16101420

**Published:** 2026-10-01

**Authors:** Cigdem Gungormez, Hatice Gumushan Aktas, Zeynep Celik, Busra Ergin, Elif Celik, Halwest Rasool Smail, Hidir Sulak

**Affiliations:** 1 Department of Medical Biology, Faculty of Medicine, Siirt University, Siirt 56100, Türkiye; 2 Biology Department, Faculty of Arts and Science, Harran University, Sanliurfa 63050, Türkiye; zeynepcelik@harran.edu.tr (Z.C.); busratosun@harran.edu.tr (B.E.); metenejat1@gmail.com (E.C.); 3 Biology Department, College of Science, University of Raparin, Raniye 46012, Iraq; halwestrasoolqanda@gmail.com; 4 Sanliurfa Youth and Sports Provincial Directorate, Sanliurfa 63210, Türkiye; dytsulak@gmail.com

**Keywords:** oleuropein, ESR2, miR-146a-5p, miR-155-5p, 5-fluorouracil, Ehrlich ascites tumor, molecular docking, breast cancer

## Abstract

Oleuropein (OL) is widely recognized for its anticancer potential, yet its impact on chemotherapy response remains insufficiently defined. This study investigated whether OL modifies the efficacy of 5-fluorouracil (5-FU) and whether these treatment responses are accompanied by changes in ESR2/ERβ expression and associated miRNA profiles in the Ehrlich ascites tumor model. OL (150 mg/kg) was administered at different treatment schedules, alone or with 5-FU (20 mg/kg), and tumor burden, ascites formation, ESR2 mRNA, ERβ protein, miR-146a-5p and miR-155-5p expression were evaluated together with validated ERβ ensemble docking. While 5-FU markedly reduced viable tumor-cell burden, concomitant OL treatment substantially weakened this effect and restored tumor-cell numbers toward untreated levels. In contrast, OL administered at tumor initiation or prophylactically reduced ascites accumulation and produced distinct ESR2/ERβ expression profiles. OL also markedly altered chemotherapy-associated microRNA responses, suppressing the strong 5-FU-induced increase in miR-146a-5p and reducing miR-155-5p after prolonged exposure. Native-ligand redocking confirmed high reliability of the ERβ docking protocol (RMSD 0.242–0.411 Å). These findings identify a treatment-dependent interplay between OL, ERβ-associated responses, and 5-FU response, revealing that OL may exert both favorable and potentially antagonistic effects depending on therapeutic context.

## 1. Introduction

Breast cancer remains a major global health burden. Updated GLOBOCAN estimates indicate that approximately 20.6 million new cancer cases and 9.8 million cancer deaths occurred worldwide in 2024; female breast cancer accounted for 11.8% of all newly diagnosed cancers and remained among the leading causes of cancer mortality [1]. Beyond its frequency, breast cancer is characterized by extensive inter- and intratumoral heterogeneity, with genomic, epigenomic, transcriptomic and microenvironmental differences contributing to variation in disease progression and therapeutic response [2].

Estrogen receptor responses is a central determinant of breast cancer biology. ERα (encoded by ESR1) is the dominant clinical biomarker and therapeutic target in hormone-receptor-positive disease, whereas ERβ (encoded by ESR2) has more context-dependent roles that vary with receptor isoform, cellular background, and ligand environment [3,4]. ERβ has frequently been associated with antiproliferative or differentiating effects, but its prognostic and functional significance cannot be generalized across breast cancer subtypes. Consequently, changes in ESR2 transcript or ERβ protein abundance should be interpreted within the accompanying molecular and treatment context rather than as stand-alone surrogates of tumor suppression [5,6,7].

MicroRNAs (miRNAs) provide an additional layer of post-transcriptional regulation in breast cancer and can influence tumor phenotype, hormone signaling, treatment response and resistance [8]. The functions of individual miRNAs are also highly context-dependent. For example, miR-146a-5p has been reported to suppress breast-cancer cell growth by targeting IRAK1 [9], but it can also support stem-like states, altered metabolism and pharmacological resistance, and has been implicated in angiogenesis and trastuzumab resistance [10,11]. Likewise, although miR-155 is often described as an oncomiR, tumor-cell-derived miR-155 can enhance antitumor immune recruitment in specific breast-cancer settings [12]. These observations underscore the need to interpret miRNA changes together with experimental context and functional endpoints [13].

Oleuropein (OL), a major secoiridoid glycoside of Olea europaea, has antioxidant, anti-inflammatory, and antineoplastic activities [14,15]. In MCF-7 breast-cancer cells, OL has been reported to inhibit proliferation and estrogen-dependent ERK1/2 signaling [16], induce cytotoxic and apoptotic responses [17], and reduce miR-155 expression [18]. However, OL can exhibit concentration- and context-dependent redox behavior; at lower exposures it may reduce oxidative stress and protect cells from certain forms of oxidative injury, whereas higher exposures can be cytotoxic [19]. This duality is particularly relevant when a phytochemical is administered together with cytotoxic chemotherapy.

The Ehrlich ascites tumor (EAT) is a rapidly growing transplantable murine mammary carcinoma-derived model widely used for in vivo anticancer screening and shares similar characteristics with human breast cancer [20]. Nevertheless, it should not be regarded as a molecular surrogate for a defined human breast-cancer subtype.

Against this background, the present study investigated whether the biological effects of OL depend on treatment schedule and chemotherapy context by evaluating viable EAT-cell burden, ascites volume, ESR2 mRNA, ERβ protein, miR-146a-5p, and miR-155-5p expression after OL alone or in combination with 5-FU. To provide complementary structural evidence, OL and oleuropein aglycone were also examined in an ensemble docking analysis against three ERβ ligand-binding domain conformations, with 17β-estradiol and genistein used as biologically relevant reference ligands and native-ligand redocking used to validate pose recovery. This study, which combines in vivo tumor-response data, ERβ-associated transcriptional and post-transcriptional signatures, and validated structural modeling, provides an integrated framework for evaluating a potential link between OL exposure, ERβ-related miRNA responses, and 5-FU responsiveness, while avoiding unsupported assumptions of a direct causal miRNA-ESR2 regulatory mechanism.

## 2. Materials and Methods

### 2.1. Experimental Design and Animals

**Figure 1:**
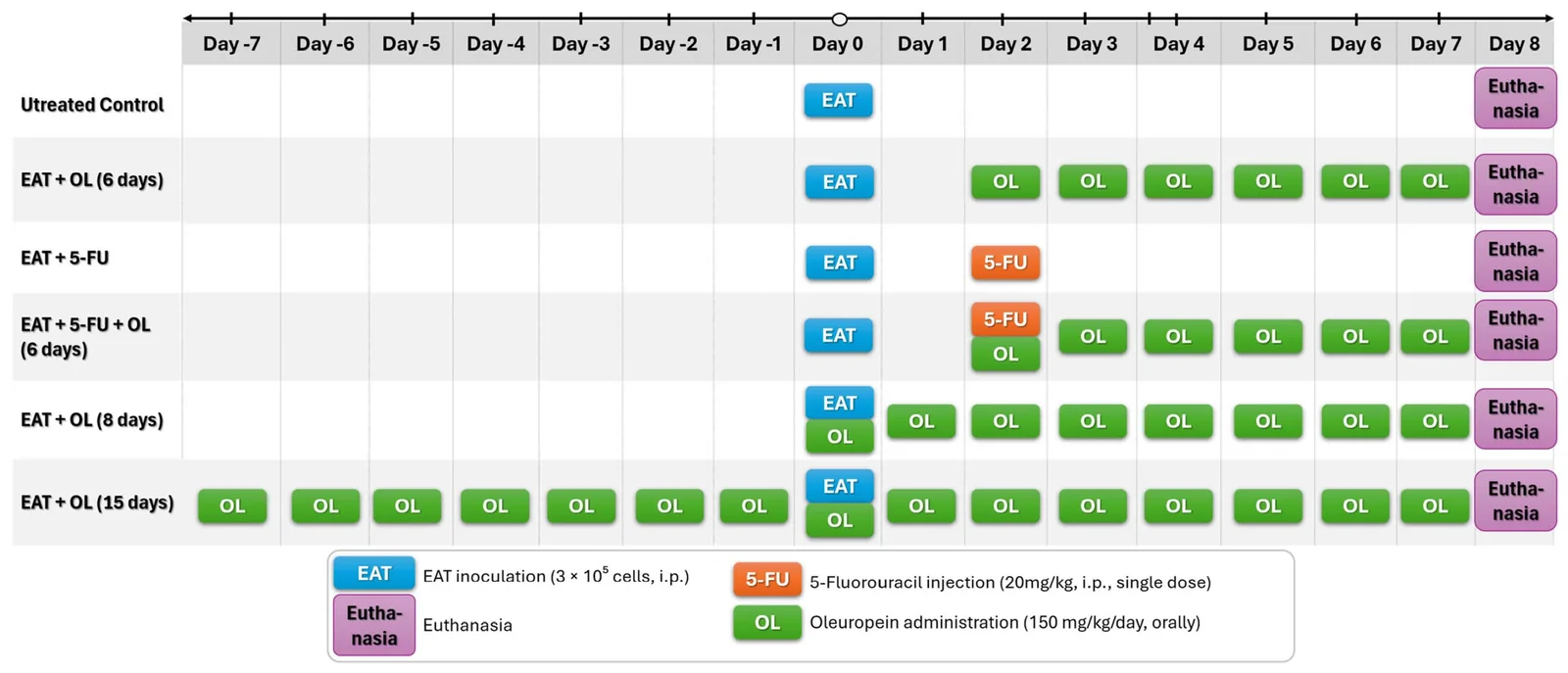
Experimental treatment protocol (n = 7 per group).

The study was conducted in 2020 following approval by the Harran University Local Animal Experiment Ethics Committee (Decision No. 2019/002/02). Inbred male BALB/c mice (approximately 2.5 months old; mean body weight, 25 g) were provided by the Experimental Animal Research Unit of Harran University, Şanlıurfa, Türkiye. The animals housed at 22 ± 2 °C and 50–60% relative humidity under a 12 h light/dark cycle with food and water available ad libitum. Male mice were preferred to minimize variability in estrogen receptor-related outcomes by avoiding fluctuations in endogenous ovarian hormones. Six experimental groups were formed (n = 7 mice/group). Ehrlich ascites tumor (EAT) cells were kindly provided by the Aziz Sancar Institute of Experimental Medicine (DETAE), Istanbul University, Istanbul, Türkiye. All animals were inoculated intraperitoneally with 3 × 10^5^ Ehrlich ascites tumor (EAT) cells on day 0. The untreated EAT control received no pharmacological intervention. The treatment schedules were designed to distinguish OL (catalog no. sc-286622B; Santa Cruz Biotechnology, Inc., Dallas, TX, USA) exposure after tumor establishment, from the day of tumor inoculation, and before tumor inoculation (Figure 1). For the tumor-growth protocol, 5-FU (20 mg/kg) was administered as a single intraperitoneal dose on day 2 to the EAT + 5-FU and EAT + 5-FU + OL groups, consistent with previously reported in vivo dosing in the EAT model [21]. OL (150 mg/kg) was administered orally by gastric gavage once daily for 6 days beginning on day 2 in the EAT + OL (6 days) and EAT + 5-FU + OL (6 days) groups. The selected OL dose was well below the previously reported oral dose of 1000 mg/kg/day administered to mice for 7 days without observed toxic effects [22]. 5-FU was administered as the commercially available injectable formulation (50 mg/mL; Koçak Farma, Istanbul, Türkiye) without an additional diluent or separately prepared vehicle. OL was dissolved in distilled water immediately before oral gavage. Food and drinking water were available ad libitum throughout the 3 experiments. For the 15-day OL pretreatment protocol, OL was initiated 7 days before EAT inoculation (Day −7) and continued through Day 7 at the same daily dose, resulting in a total OL exposure period of 15 days.

### 2.2. Collection of Ascitic Fluid and Assessment of Viable Tumor-Cell Burden

On Day 8 after EAT inoculation, ascitic fluid containing EAT cells was collected from the peritoneal cavity under ketamine/xylazine anesthesia in all experimental groups. Thus, all tumor-bearing animals had the same duration of tumor growth, and the 15-day protocol refers to the total duration of OL exposure (Day −7 to Day 7), rather than to a later experimental endpoint. Total ascites volume was recorded, and viable EAT-cell density was determined by Trypan blue exclusion [23]. Total viable tumor-cell burden was calculated from viable-cell density and recovered ascites volume. After completion of the experimental procedures, animals were euthanized in accordance with the approved ethical protocol. EAT cells isolated from peritoneal fluid were washed with phosphate-buffered saline (PBS), divided into aliquots and cryopreserved in liquid nitrogen for subsequent molecular analyses.

### 2.3. Selection of Candidate microRNAs

Candidate miRNAs were prioritized using an integrative, hypothesis-generating in silico workflow. Putative miRNAs targeting the human ESR2 3′-untranslated region were retrieved from TargetScan Human Release 8.0 and considered in relation to predicted context++ scores and site conservation [24]. Publicly available human breast-cancer miRNA expression datasets were then examined using GEO2R/limma with Benjamini–Hochberg adjustment [25]. GSE35412 was used as the primary discovery dataset and GSE45666, GSE42128 and GSE38167 as additional datasets. Differential expression was defined using adjusted *p* < 0.05 and |log2 fold change| ≥ 1. miR-146a-5p and miR-155-5p were selected on the basis of predicted ESR2 relevance, expression patterns and prior breast-cancer biology. This bioinformatic workflow prioritized candidate miRNAs rather than direct miRNA–ESR2 interactions. Given the human-based predictions and murine experimental model, the observed ESR2/ERβ–miRNA relationships were interpreted as hypothesis-generating associations.

### 2.4. mRNA and miRNA Expression Analysis

Total RNA, including small RNAs, was isolated from EAT-cell samples using the miRNeasy Mini Kit (Qiagen, Germantown, MD, USA) after lysis in QIAzol. RNA concentration and purity were assessed spectrophotometrically. For miRNA analysis, cDNA was generated using the miScript II RT Kit (Qiagen), and qPCR was performed using the miScript SYBR Green PCR Kit (Qiagen, Germantown, MD, USA) on a Bio-Rad real-time PCR platform(Bio-Rad Laboratories, Hercules, CA, USA). Cycling consisted of 95 °C for 15 s, 55 °C for 30 s and 70 °C for 30 s per cycle. SNORD65 was used as the reference small RNA, and relative expression was calculated by the 2^−ΔΔCt^ method [26].

For ESR2 mRNA analysis, total RNA was reverse-transcribed using the High-Capacity cDNA Reverse Transcription Kit (Thermo Fisher Scientific, Waltham, MA, USA). qPCR reactions were prepared using SYBR Green Master Mix (Qiagen, Germantown, MD, USA) and analyzed on a Bio-Rad CFX96 system. The protocol comprised an initial denaturation at 95 °C for 3 min followed by 40 cycles including denaturation at 95 °C for 15 s and primer annealing at 63 °C for 1 min. GAPDH (forward: 5′-CCCAGCTTAGGTTCATCAGG-3′ and reverse: 5′-TGATGTTAGTGGGGTCTCGC-3′) was used as the reference gene, and relative ESR2 (forward: 5′-GACGAAGAGTGCTGTCCCAA-3′ and reverse: 5′-TCAGCTTCCGGCTACTCTCT-3′) expression was calculated by the 2^−ΔΔCt^ method.

All relative-expression values are presented as fold change (FC) relative to the specified control group. The experimental interpretation focuses on group-level expression responses; direct miRNA binding to the ESR2/Esr2 3′-UTR was not tested in this study.

### 2.5. Protein Isolation and Western Blot Analysis

For protein extraction, equal numbers of EAT cells (1 × 10^6^) were washed with PBS and lysed in RIPA buffer containing protease/phosphatase inhibitors (SantaCruz Biotechnology, RIPA Lysis Buffer System, (#sc-24948A; Santa Cruz Biotechnology, Dallas, TX, USA). Western blot analyses were performed using three independent biological replicates per group (n = 3), with each replicate representing an individual animal. Lysates were clarified by centrifugation (14,000 rpm, 4 °C, 15 min), and protein concentration was determined by the Bradford method [27]. Equal protein amounts (30 µg) were denatured in Laemmli buffer, separated by SDS-PAGE and transferred to PVDF membranes using a Bio-Rad Trans-Blot Turbo system. Membranes were blocked in 5% skim milk/TBST for 1 h and incubated overnight at 4 °C with primary antibodies against ERβ (approximately 59 kDa, Affinity Biosciences, #AF6469, Cincinnati, OH, USA) and β-actin (approximately 42 kDa, #sc-47778, Santa Cruz Biotechnology, Dallas, TX, USA), followed by the appropriate secondary antibody. Chemiluminescent signals were recorded using a ChemiDoc MP system (Bio-Rad Laboratories, Inc., Hercules, CA, USA) and quantified with Image Lab software. (version 6.0.1; Bio-Rad Laboratories, Inc., Hercules, CA, USA) ERβ band intensity was normalized to β-actin.

### 2.6. Molecular Docking and Redocking Validation

The crystallographic structures of the human ERβ ligand-binding domain were obtained from the RCSB Protein Data Bank: 1QKM, 3OLS and 5TOA. Chain A was retained from each structure. The co-crystallized ligand, crystallographic water molecules and other non-protein heteroatoms were removed before docking. The receptors were treated as rigid structures. No separate receptor energy-minimization procedure was performed. Missing side-chain atoms and hydrogen atoms were added automatically by the CB-Dock2 preparation pipeline; missing backbone residues were not reconstructed [28].

Three-dimensional ligand structures were obtained from PubChem in SDF format: oleuropein (CID 5281544), oleuropein aglycone (CID 56842347), genistein (CID 5280961) and 17β-estradiol (CID 5757). The stereochemical configurations encoded in the PubChem records were retained. No alternative stereoisomers were generated. The ligands were submitted in their neutral forms, without manual assignment of alternative pH-dependent protonation states. 17β-Estradiol was included as the endogenous ERβ reference ligand, whereas genistein served as an ERβ-preferring phytoestrogen kuireference [29]. Although oleuropein aglycone was not administered in the in vivo experiments, it was included as a structurally and biologically relevant comparator because it represents the deglycosylated form of oleuropein and has been implicated as a bioactive derivative generated during oleuropein processing and metabolism. CB-Dock2 automatically added hydrogen atoms and partial charges and generated the initial three-dimensional ligand conformations using RDKit. No additional external ligand-minimization procedure was applied.

Docking was performed using the CB-Dock2 web server, as described by Liu et al. (2022), accessed on [4 August 2026] [30]. CB-Dock2 combines structure-based cavity detection with AutoDock Vina (version 1.2.0) -based docking and a homologous-template-based FitDock pathway. The default setting of five predicted cavities was used. For each receptor, the cavity corresponding to the canonical ERβ ligand-binding region was selected on the basis of its spatial overlap with the position of the removed crystallographic ligand. The highest-ranked pose within this predefined ligand-binding region was retained for descriptive comparison. Docking scores were interpreted as relative computational scores and not as experimentally determined binding free energies.

For the pose-recovery assessment, genistein was redocked into ligand-free 1QKM, whereas 17β-estradiol was redocked into ligand-free 3OLS and 5TOA. Heavy-atom RMSD calculations were performed using RDKit (version 2025.09.4) in Python 3.13.5, with numerical operations carried out using NumPy (version 2.3.5) [31]. Atom correspondence between the crystallographic and redocked ligands was established on the basis of molecular topology, and RMSD values were calculated directly in the unchanged receptor coordinate frame without prior ligand fitting or superposition. An RMSD below 2.0 Å was considered successful pose recovery [32]. However, because CB-Dock2 searches a BioLiP-derived database and can integrate homologous-template information into cavity and pose prediction, the native or related ligand-bound structures may have influenced the redocking results. The RMSD values are therefore presented as an internal pose-recovery assessment within the CB-Dock2 workflow and not as independent validation of the method.

### 2.7. Statistical Analysis

Quantitative data are presented as mean ± standard error of the mean (SEM). Differences among experimental groups were assessed using one-way analysis of variance (ANOVA), followed by Tukey’s honestly significant difference (HSD) or least significant difference (LSD) post hoc tests for multiple comparisons, as appropriate. Student’s *t*-test was used for selected pairwise comparisons. Statistical analyses and data processing were performed using SPSS (IBM SPSS Statistics, version 22.0) and Microsoft Excel. A two-sided *p*-value < 0.05 was considered statistically significant.

## 3. Results and Discussion

### 3.1. Treatment-Context-Dependent Effects of Oleuropein on Viable EAT-Cell Burden and Ascites Formation

**Figure 2:**
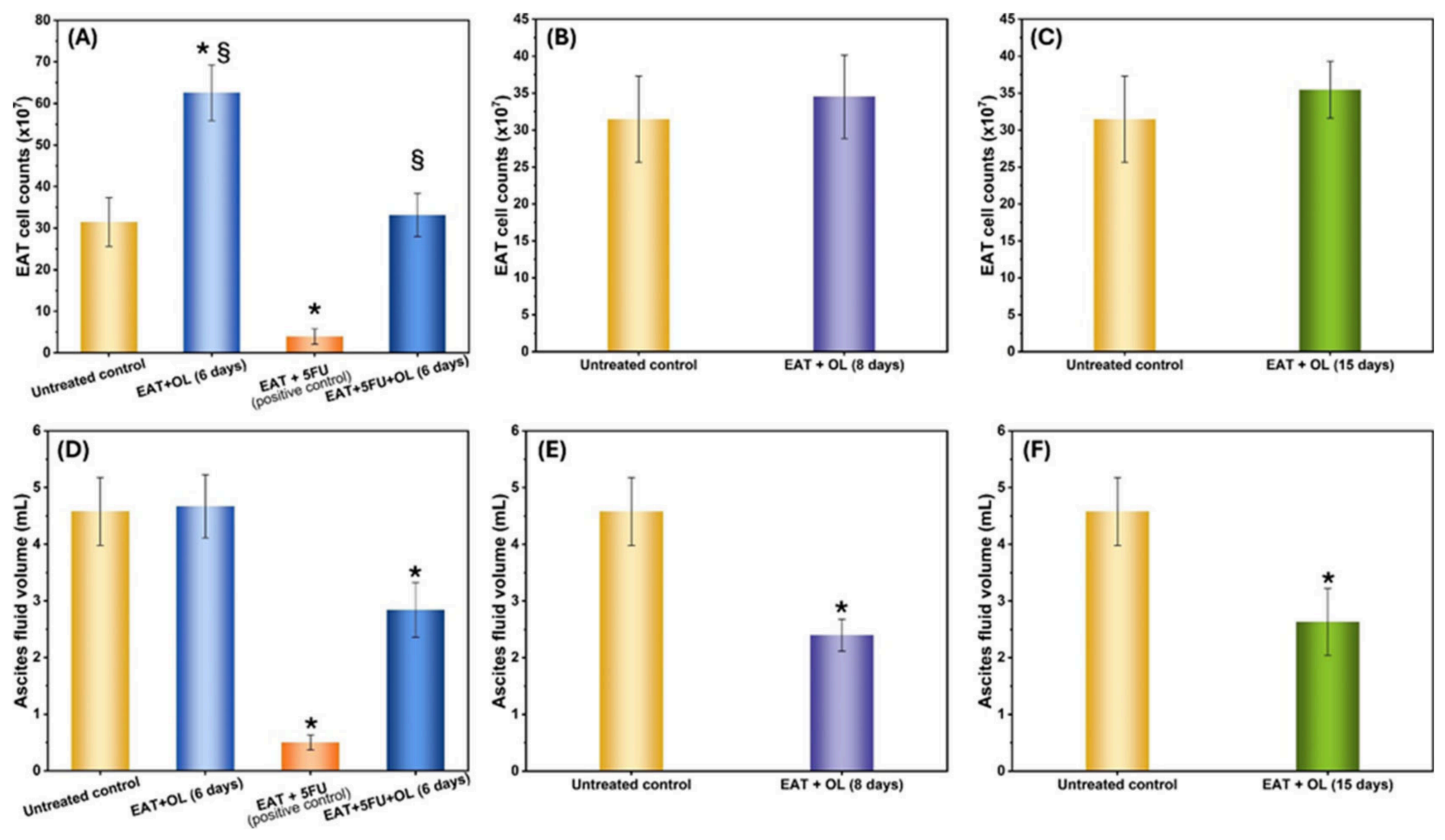
Effects of oleuropein (OL) and 5-fluorouracil (5-FU) on (**A**–**C**) total viable EAT-cell burden and (**D**–**F**) ascites fluid volume across the 6-, 8- and 15-day treatment schedules. Data are mean ± SEM. * *p* < 0.05 vs. untreated EAT control; § *p* < 0.05 vs. EAT + 5-FU.

Treatment responses differed markedly according to schedule (Table 1; Figure 2). Untreated EAT controls contained 31.48 ± 5.83 × 10^7^ viable tumor cells. OL administered for 6 days after tumor establishment increased viable EAT-cell burden to 62.59 ± 6.70 × 10^7^ cells, representing an approximately 98.8% increase relative to control (*p* < 0.05). The increase in viable tumor-cell burden observed with the 6-day OL regimen highlights the importance of treatment timing in determining the biological response to OL in the EAT model. This effect may reflect shifts in the balance between tumor-cell proliferation, survival, and host-mediated clearance. In contrast, the reduction in ascites with earlier OL administration suggests that the biological effects of OL vary according to the stage and timing of treatment. By contrast, a single dose of 5-FU reduced viable tumor-cell burden to 3.96 ± 1.85 × 10^7^ cells (approximately 87.4% below control; *p* < 0.05). When OL was co-administered with 5-FU, viable cell burden increased to 33.21 ± 5.21 × 10^7^ cells and was significantly higher than with 5-FU alone (*p* < 0.05), while no significant difference from untreated control was indicated. Thus, under the present schedule, OL did not potentiate 5-FU cytotoxicity; rather, it attenuated the 5-FU-associated reduction in viable tumor cells. This finding contrasts with in vitro reports in which OL inhibited MCF-7 proliferation or induced cytotoxicity [16,17], but it is compatible with evidence that OL can exert dose- and context-dependent antioxidant versus pro-oxidant actions [19]. Recent in vivo studies further demonstrate that OL can attenuate 5-FU-induced nephrohepatic, cardiac, and pulmonary injury through antioxidant, anti-inflammatory, and anti-apoptotic mechanisms [16,17]. Importantly, such protection against chemotherapy-associated injury in non-tumor tissues should be distinguished from effects on antitumor efficacy. In the present EAT model, concomitant OL administration attenuated the 5-FU-associated reduction in viable tumor-cell burden, indicating that the biological consequences of OL–5-FU co-administration may differ between normal-tissue protection and tumor response. The present findings therefore suggest that OL may modulate the cellular response to 5-FU rather than acting as a uniformly synergistic anticancer agent, underscoring the need to consider treatment timing and combination strategy when evaluating its therapeutic potential.

Ascites volume showed a partially dissociated response. 5-FU reduced ascites from 4.58 ± 0.60 to 0.50 ± 0.13 mL (approximately 89.1%; *p* < 0.05). The 5-FU+OL group also had lower ascites volume than the untreated control (2.84 ± 0.48 mL; approximately 38.0% reduction; *p* < 0.05), despite recovery of viable cell burden toward the control value. Likewise, OL administered for 8 or 15 days reduced ascites volume to 2.40 ± 0.28 and 2.63 ± 0.59 mL, respectively (approximately 47.6% and 42.6% below control; both *p* < 0.05), without a significant reduction in viable tumor-cell number. These findings indicate that ascites accumulation and viable EAT-cell burden reflect related but distinct aspects of tumor progression. Accordingly, the reduction in fluid accumulation observed in early and prophylactic OL programs, while not direct evidence of tumor cell cytotoxicity, may represent a beneficial effect on the tumor microenvironment and disease-related fluid dynamics.

### 3.2. Oleuropein Modifies ESR2/ERβ Responses During Established Tumor Growth and 5-FU Treatment

**Figure 3:**
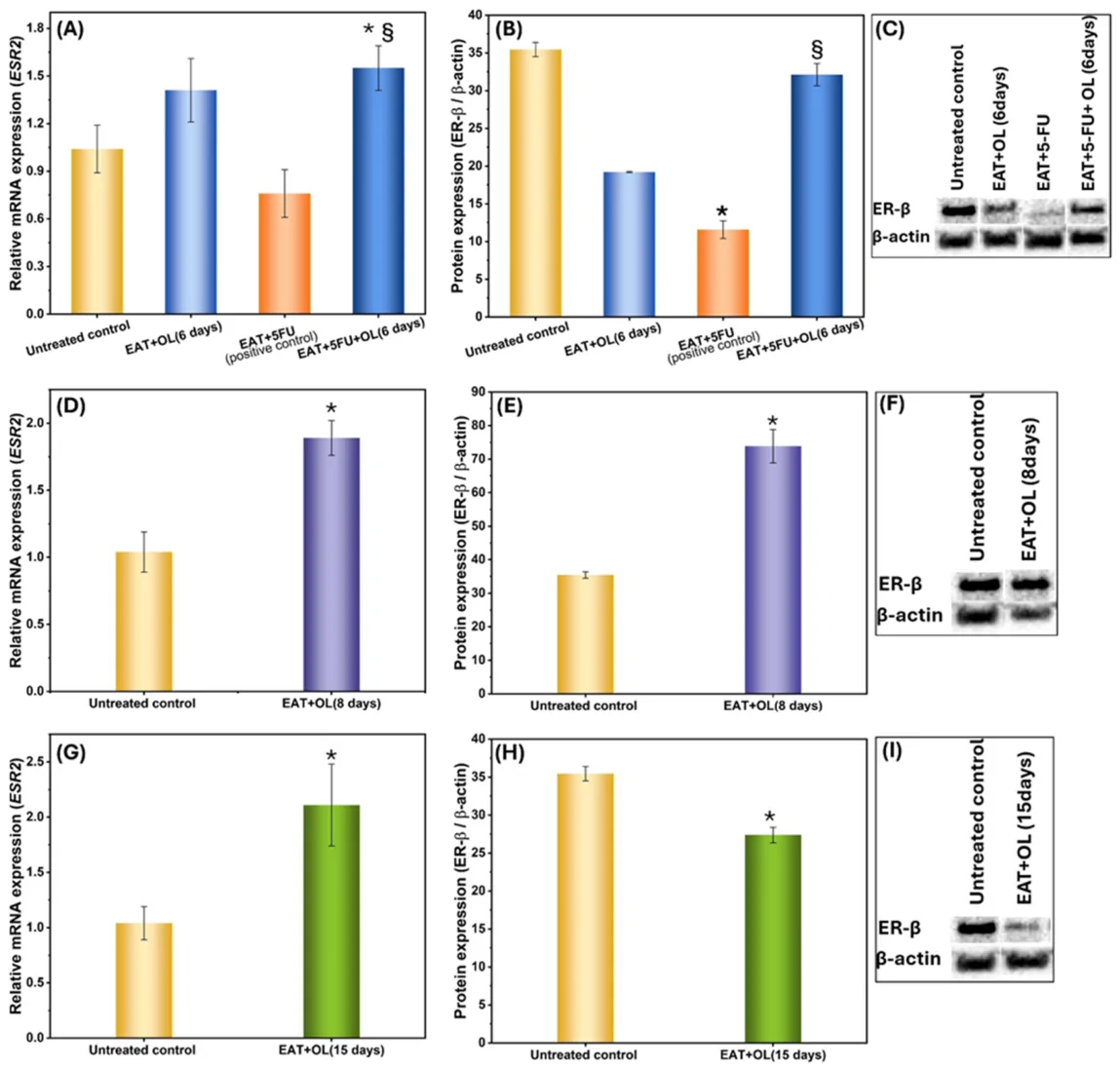
ESR2/ERβ responses to oleuropein (OL) and 5-fluorouracil (5-FU). ESR2 mRNA expression is shown in panels (**A**,**D**,**G**); ERβ protein quantification in (**B**,**E**,**H**); and representative ERβ/β-actin Western blots in (**C**,**F**,**I**) for the 6-, 8- and 15-day protocols, respectively. Data are mean ± SEM. * *p* < 0.05 vs. untreated EAT control; § *p* < 0.05 vs. EAT + 5-FU. Original Western Blot images can be found in Supplementary Materials.

During established tumor growth, ESR2 mRNA expression was 1.04 ± 0.15 FC in untreated controls, 1.41 ± 0.20 after 6-day OL treatment and 0.76 ± 0.15 after 5-FU. Combined 5-FU + OL increased ESR2 mRNA to 1.55 ± 0.14 FC, significantly above both untreated control and 5-FU (*p* < 0.05; Table 2; Figure 3A). ERβ protein expression showed a different magnitude of response: 35.44 ± 0.95% in controls, 19.21 ± 0.08% after 6-day OL, 11.57 ± 1.17% after 5-FU (*p* < 0.05 vs. control), and 32.11 ± 1.46% after combined treatment (*p* < 0.05 vs. 5-FU; Table 2; Figure 3B,C). Thus, OL co-administration largely restored ERβ protein abundance relative to 5-FU alone while increasing ESR2 transcript abundance.

ERβ signaling in breast cancer is strongly dependent on receptor isoform, tumor subtype, ligand environment and cellular context [3,4] Interestingly, OL co-treatment restored ESR2/ERβ expression toward or above control levels while simultaneously attenuating the marked reduction in viable tumor-cell burden produced by 5-FU alone. This dissociation indicates that increased ERβ expression was not directly coupled to tumor suppression under the present experimental conditions. Rather, the findings suggest that OL-induced modulation of ESR2/ERβ occurs within a broader, treatment-dependent molecular response that may involve additional regulatory pathways.

### 3.3. Schedule-Dependent Coupling of ESR2 mRNA and ERβ Protein During Early and Prophylactic OL Exposure

When OL administration was initiated on the day of EAT inoculation and continued for 8 days, ESR2 mRNA increased from 1.04 ± 0.15 to 1.89 ± 0.13 FC (*p* < 0.05), accompanied by a pronounced rise in ERβ protein from 35.44 ± 0.95% to 73.88 ± 4.97% (*p* < 0.05; Table 2; Figure 3D–F). This concordant increase at both transcript and protein levels indicates that early OL exposure can strongly activate the ESR2/ERβ axis during tumor establishment. Importantly, this molecular response coincided with a significant reduction in ascites volume, suggesting that ERβ upregulation may be associated with changes in the ascitic tumor microenvironment and disease progression, even when the number of viable tumor cells is not significantly altered.

A different regulatory pattern emerged in the prophylactic 15-day protocol. ESR2 mRNA increased to 2.11 ± 0.37 FC (*p* < 0.05), whereas ERβ protein decreased to 27.38 ± 1.02% (*p* < 0.05; Table 2; Figure 3G–I). Rather than representing a simple inconsistency, this divergence highlights a shift in the regulation of the ESR2/ERβ pathway during prolonged OL exposure. The persistence of transcriptional induction together with reduced protein abundance is consistent with the involvement of post-transcriptional regulation, altered translational efficiency, receptor turnover, or feedback mechanisms controlling ERβ availability. However, because these mechanisms were not directly measured, the discrepancy is best treated as a mechanistic lead. This schedule-dependent transition from coordinated mRNA-protein upregulation at 8 days to transcript–protein dissociation at 15 days provides evidence that OL does not regulate ESR2/ERβ in a static manner, but instead induces temporally dynamic receptor regulation. These findings strengthen the view that treatment timing is a critical determinant of OL-mediated estrogen receptor signaling in the EAT model.

### 3.4. miR-146a-5p Reveals a Prominent 5-FU-Associated Response That Is Attenuated by OL

**Figure 4:**
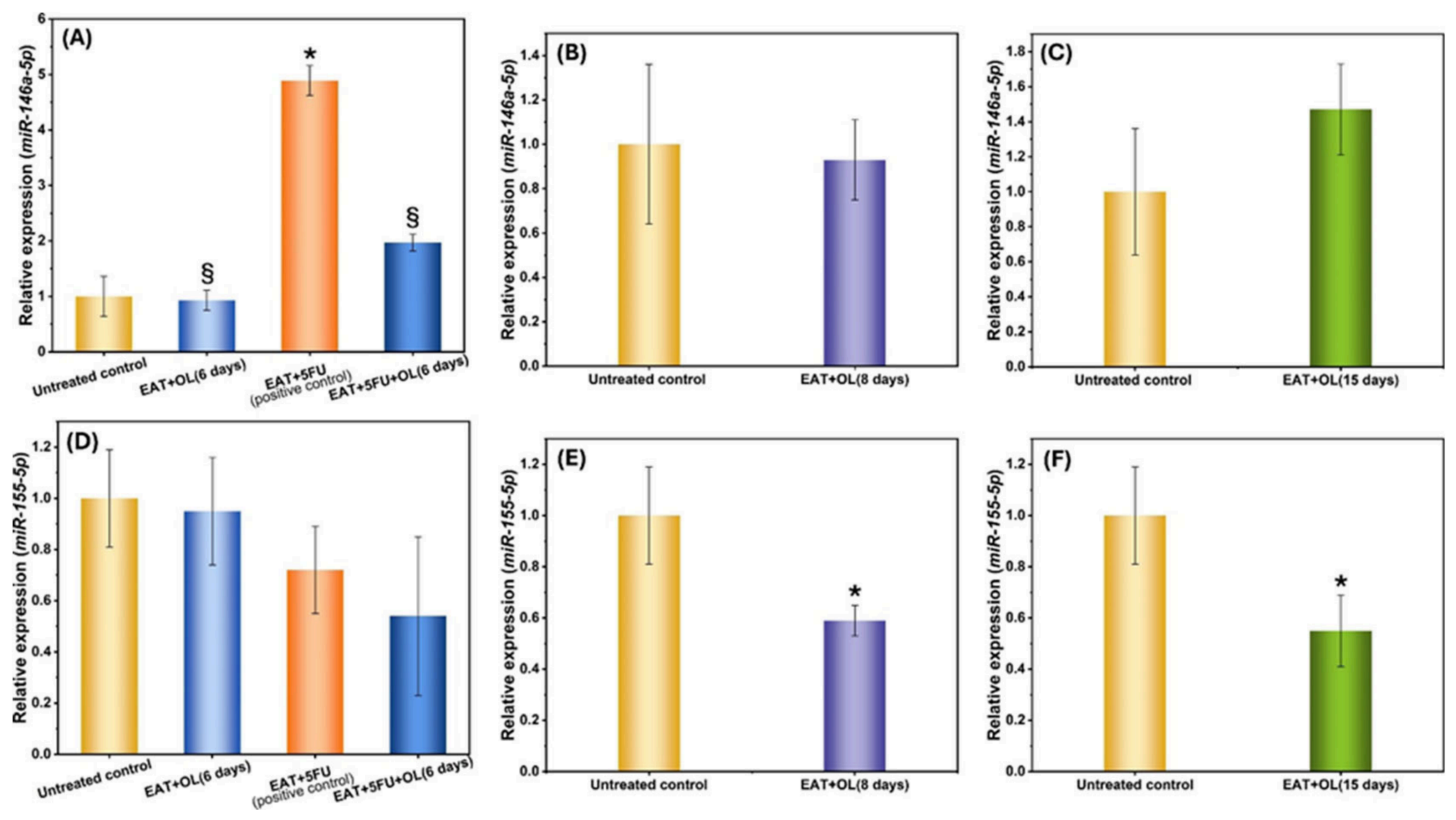
Relative expression of (**A**–**C**) miR-146a-5p and (**D**–**F**) miR-155-5p after oleuropein (OL) and/or 5-fluorouracil (5-FU) treatment across the 6-, 8- and 15-day protocols. Data are mean ± SEM. * *p* < 0.05 vs. untreated EAT control; § *p* < 0.05 vs. EAT + 5-FU.

miR-146a-5p expression was 1.00 ± 0.36 FC in untreated controls and 0.93 ± 0.18 after 6-day OL treatment. The strongest response occurred after 5-FU, which increased miR-146a-5p to 4.89 ± 0.27 FC (*p* < 0.05 vs. control). Co-administration of OL reduced this 5-FU-associated increase to 1.97 ± 0.15 FC (*p* < 0.05 vs. 5-FU), while the 8- and 15-day OL groups showed more modest, non-significant values of 1.43 ± 0.15 and 1.47 ± 0.26 FC (Table 3; Figure 4A–C). The biological meaning of the 5-FU-induced miR-146a-5p response is not necessarily unidirectional. miR-146a-5p can suppress breast cancer cell growth by targeting IRAK1 [9], yet other work links miR-146 family activity to stem-like states, antifolate resistance, angiogenesis, and trastuzumab resistance [10,11]. The present data therefore demonstrate that OL modulates a chemotherapy-responsive miRNA but do not establish whether that modulation is beneficial or detrimental without direct functional testing.

### 3.5. Prolonged OL Exposure Reduces miR-155-5p Expression

miR-155-5p expression was 1.00 ± 0.19 FC in untreated controls, 0.95 ± 0.21 after 6-day OL, 0.72 ± 0.16 after 5-FU and 0.54 ± 0.31 after combined 5-FU + OL treatment. Importantly, the 8- and 15-day OL protocols reduced miR-155-5p to 0.59 ± 0.06 and 0.55 ± 0.14 FC, respectively, and both decreases were statistically significant relative to untreated control (*p* < 0.05; Table 3; Figure 4D–F). This pattern is consistent with an earlier MCF-7 study in which OL reduced miR-155 expression in a time- and dose-dependent manner [18]. Nevertheless, miR-155 should not be interpreted as uniformly oncogenic: breast-cancer-cell-derived miR-155 can also enhance antitumor immune recruitment in specific settings [12]. Thus, the observed decrease is a robust molecular response to prolonged OL exposure in the present EAT model, but its functional consequence remains to be established.

### 3.6. Integrated Interpretation of ESR2/ERβ and miRNA Responses

The combined molecular profiles reveal a treatment-dependent relationship between ESR2/ERβ responses and miRNA expression rather than a uniform linear regulatory pattern across all groups. The contrast between 5-FU and 5-FU+OL is particularly informative: strong miR-146a-5p induction after 5-FU coincided with low ERβ protein expression, whereas OL co-treatment reduced miR-146a-5p expression and restored ERβ abundance toward control levels. This reciprocal pattern supports a potential functional association between miR-146a-5p and ERβ regulation during chemotherapy exposure. In contrast, the 15-day OL group showed increased ESR2 mRNA together with reduced ERβ protein, despite significant suppression of miR-155-5p and no significant change in miR-146a-5p. This divergence indicates that prolonged OL exposure engages additional layers of post-transcriptional and protein-level regulation beyond the two miRNAs examined. Taken together, these findings demonstrate schedule-dependent changes in ESR2/ERβ and miRNA expression following OL treatment, with miR-146a-5p showing the most prominent response in the 5-FU treatment context. Further functional validation using species-appropriate reporter assays, miRNA mimic/inhibitor approaches, and rescue experiments would help define the direct contribution of these miRNAs to ESR2 regulation.

### 3.7. Ensemble Docking Indicates Conformation-Dependent Structural Compatibility of Oleuropein with ERβ Models

To investigate whether the treatment-associated changes in ESR2/ERβ observed in vivo could be supported by a plausible structural interaction between oleuropein (OL) and ERβ, OL was docked against three experimentally determined ERβ ligand-binding-domain conformations (1QKM, 3OLS, and 5TOA). The reliability of the docking workflow was first confirmed by native-ligand redocking. The crystallographic ligands were reproduced with heavy-atom RMSD values of 0.242 Å for genistein in 1QKM and 0.411 and 0.356 Å for 17β-estradiol in 3OLS and 5TOA, respectively (Table 4). These values were well below the 2.0 Å criterion used for successful pose recovery in the CB-Dock2 benchmark, demonstrating that the docking protocol accurately reproduced the experimentally observed ERβ ligand-binding geometries [33].

**Figure 5:**
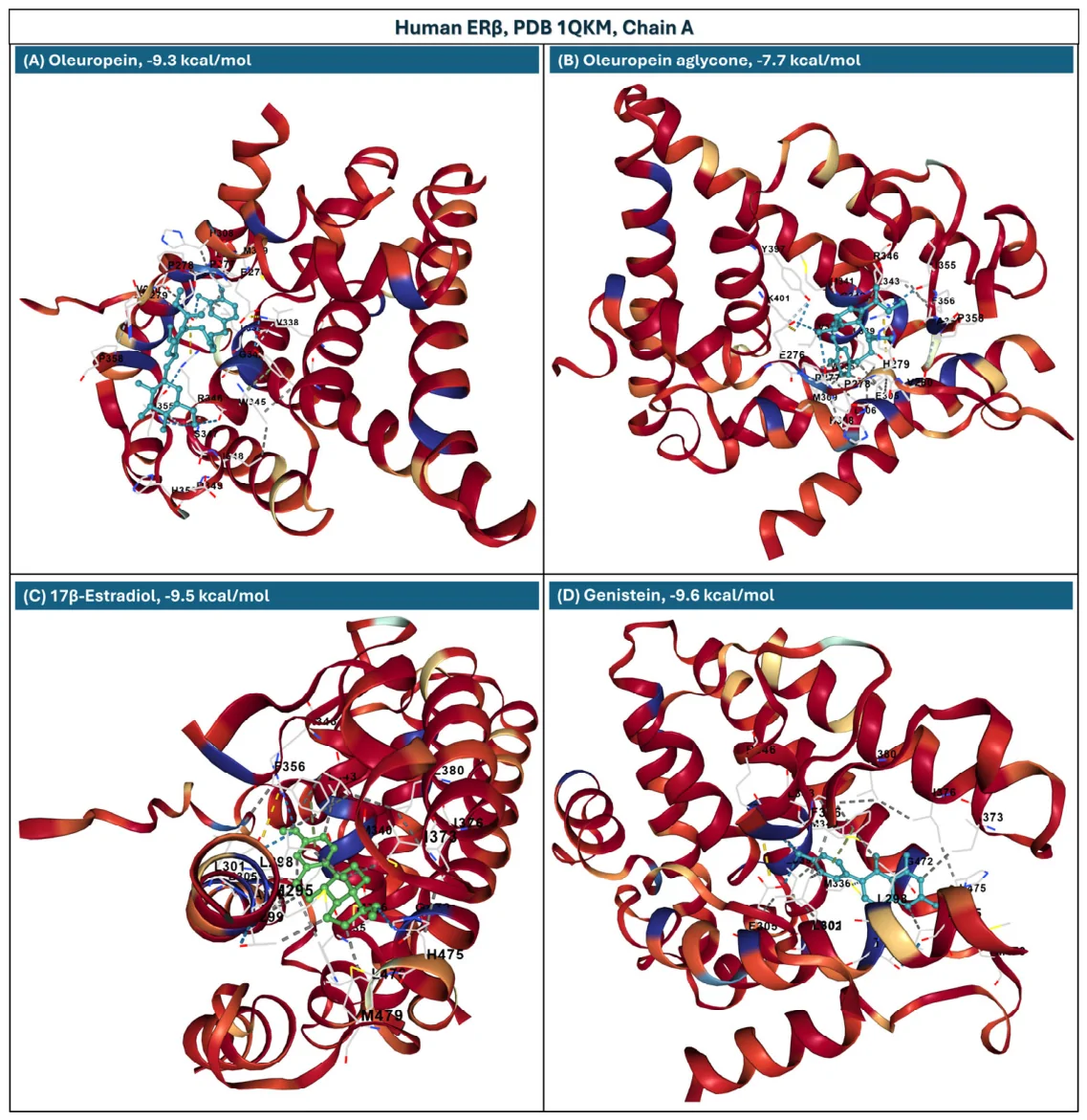
Predicted docking poses of the top-ranked ligands within the ligand-binding pocket of human ERβ, PDB ID 1QKM, chain A: (**A**) oleuropein, −9.3 kcal/mol; (**B**) oleuropein aglycone, −7.7 kcal/mol; (**C**) 17β-estradiol, −9.5 kcal/mol; and (**D**) genistein, −9.6 kcal/mol. The receptor is shown as a cartoon, and the ligands and contacting residues are shown as sticks. Labeled residues indicate amino acids predicted to contact the ligand. Dashed lines represent predicted non-covalent contacts, and the associated values indicate atom-to-atom distances in Å. All panels show the top-ranked pose within the selected ERβ ligand-binding cavity and are presented using the same receptor orientation. Images were rendered using the NGL Viewer integrated into CB-Dock2. These structures are computationally predicted docking poses and not experimentally determined OL–ERβ complexes.

**Figure 6:**
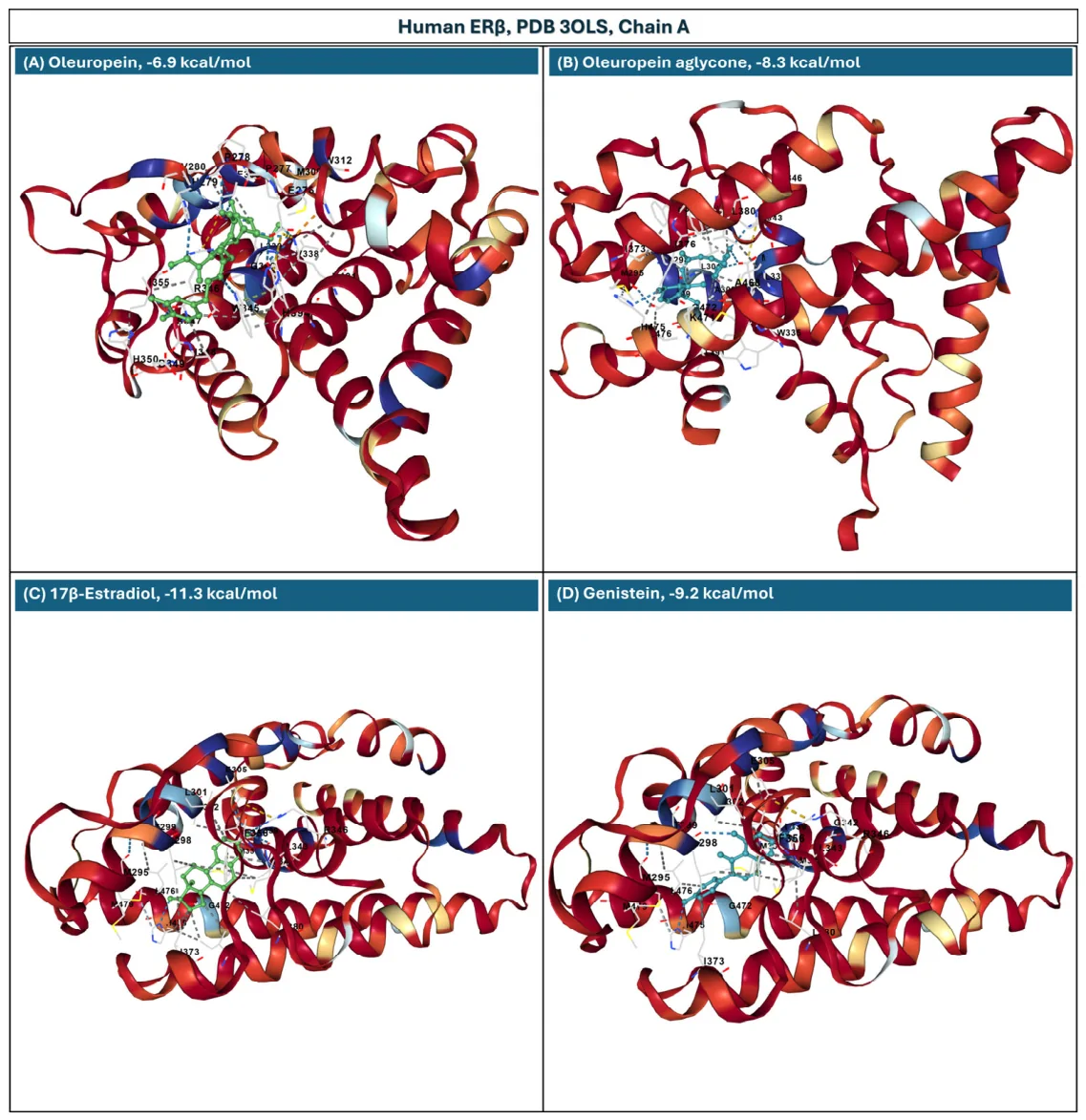
Predicted docking poses of the top-ranked ligands within the ligand-binding pocket of human ERβ, PDB ID 3OLS, chain A: (**A**) oleuropein, −6.9 kcal/mol; (**B**) oleuropein aglycone, −8.3 kcal/mol; (**C**) 17β-estradiol, −11.3 kcal/mol; and (**D**) genistein, −9.2 kcal/mol. Representations, residue labels, contact lines, distance values, receptor orientation, and rendering software are as described for Figure 5. These structures are computationally predicted docking poses and not experimentally determined ligand–ERβ complexes.

OL showed favorable but receptor-conformation-dependent binding across the ERβ ensemble. Its strongest interaction was predicted for 1QKM, with a binding score of −9.3 kcal/mol, closely approaching those of the ERβ-preferring phytoestrogen genistein (−9.6 kcal/mol) and the endogenous ligand 17β-estradiol (−9.5 kcal/mol). In the estradiol-bound ERβ structures, OL yielded scores of −6.9 kcal/mol for 3OLS and −8.0 kcal/mol for 5TOA (Table 4, Figure 5 and Figure 6 and Supplementary File). Thus, the parent compound showed its most favorable accommodation in the 1QKM ligand-binding domain, whereas its interaction was weaker in the alternative ERβ conformations. The representative poses shown in Figure 5 and Figure 6 further demonstrate that OL can be accommodated within the ERβ ligand-binding region despite its relatively large and glycosylated structure. Taken together, these findings indicate that OL possesses structural compatibility with ERβ, but that the strength of this interaction is influenced by the conformational state of the receptor.

The behavior of the reference ligands provided additional biological context for interpreting the OL results. Genistein, a well-established ERβ-preferring phytoestrogen, showed its most favorable score in the genistein-bound 1QKM structure (−9.6 kcal/mol), whereas 17β-estradiol exhibited the strongest interactions with the estradiol-associated 3OLS and 5TOA conformations (−11.3 and −11.1 kcal/mol, respectively) [29,30]. This correspondence between the experimentally bound ligands and their preferred receptor templates further supports the ability of the ensemble approach to capture ligand-dependent ERβ conformational preferences.

Oleuropein aglycone was included as a secondary structural comparator to assess whether removal of the glycosyl moiety altered ERβ accommodation. Its binding scores were −7.7, −8.3, and −8.0 kcal/mol for 1QKM, 3OLS, and 5TOA, respectively. Deglycosylation therefore did not produce a uniformly stronger interaction than the parent compound: OL was more favorable in 1QKM, the aglycone was more favorable in 3OLS, and both compounds produced the same score in 5TOA. However, the aglycone showed substantially less variation across the three receptor conformations, with a score range of only 0.6 kcal/mol compared with 2.4 kcal/mol for OL. This suggests that the smaller aglycone can be accommodated more consistently across different ERβ conformations, whereas the larger glycosylated OL displays greater conformational selectivity. Recent cellular evidence indicating that oleuropein aglycone can influence ERβ-associated signaling further supports the biological relevance of including this derivative as a structural comparator [31].

**Figure 7:**
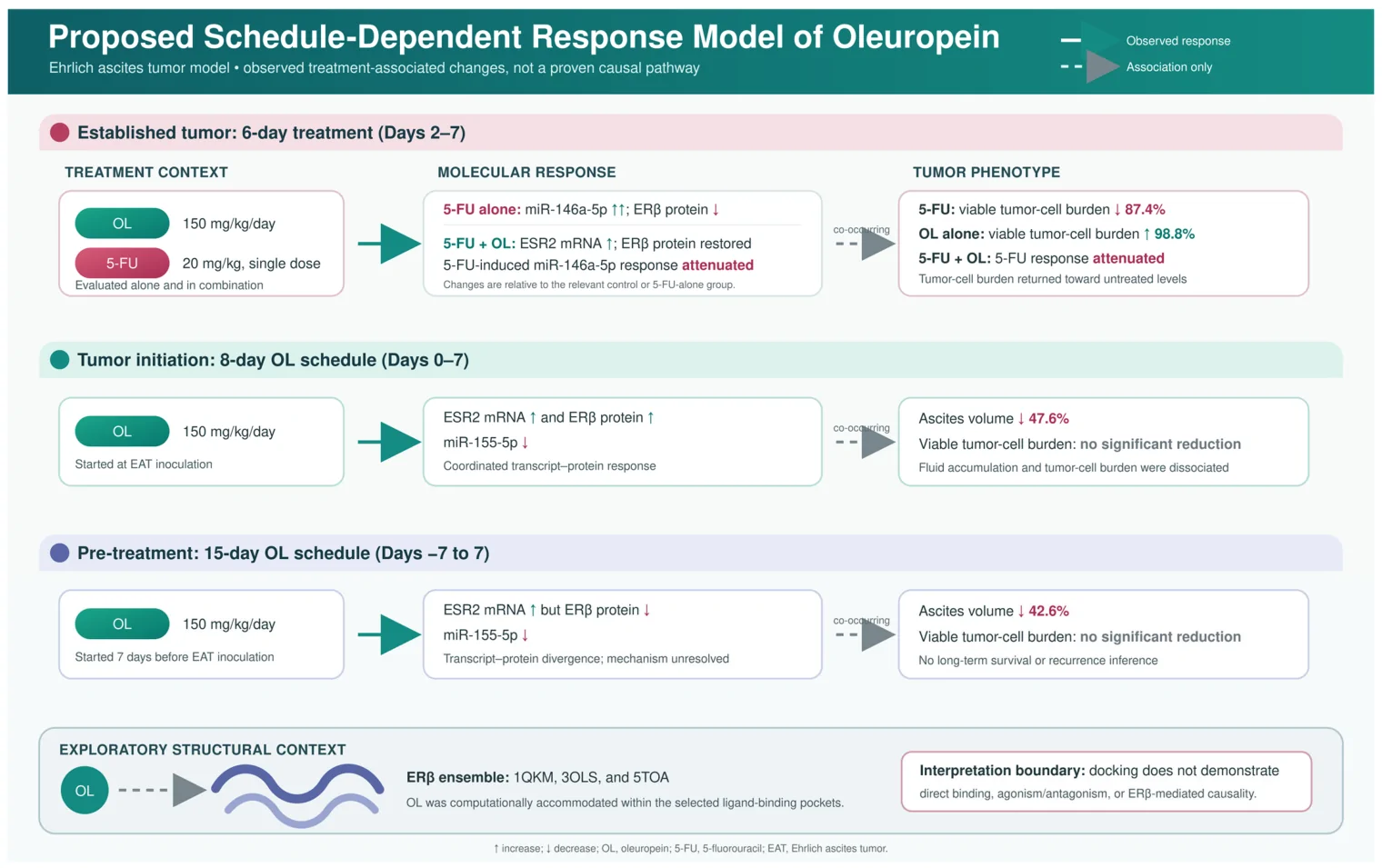
Proposed schedule-dependent response model of oleuropein in the Ehrlich ascites tumor model. The diagram summarizes the molecular and tumor-related changes observed following 6-, 8-, and 15-day OL administration schedules, alone or with 5-FU. Solid arrows represent observed treatment responses, whereas dashed arrows indicate co-occurring associations without implying causality. The docking panel shows the predicted accommodation of OL within the ligand-binding regions of the 1QKM, 3OLS, and 5TOA ERβ structures. These docking findings provide exploratory structural context but do not demonstrate direct OL–ERβ binding or ERβ-mediated activity. OL, oleuropein; 5-FU, 5-fluorouracil; ERβ, estrogen receptor beta; ESR2, estrogen receptor beta gene.

The three receptor structures used in this study represent distinct experimentally observed ERβ ligand-bound states: 1QKM contains genistein in a partial-agonist-associated conformation, 3OLS represents an estradiol-bound/coactivator-associated complex, and 5TOA contains an alternative estradiol-bound helix-12 arrangement [34,35]. Using this ensemble enabled the influence of receptor conformation on the predicted accommodation of OL to be explored. OL was computationally accommodated within the ligand-binding region of all three structures, with its most favorable docking score obtained for 1QKM. However, these predictions do not demonstrate direct OL–ERβ binding or establish ERβ as the mediator of the observed in vivo responses. Accordingly, the docking findings are interpreted as exploratory structural context for the schedule-dependent tumor and molecular responses, which are integrated in the proposed model shown in Figure 7. The figure distinguishes experimentally observed treatment-associated changes from relationships that remain hypothetical and require functional validation.

### 3.8. Strengths and Limitations

A principal strength of this study is its integrated design, combining tumor-related outcomes with molecular analyses of ESR2 mRNA, ERβ protein, and selected miRNAs, together with exploratory molecular docking across different OL treatment schedules. This approach enabled comparative evaluation of schedule-dependent responses to OL alone and in combination with 5-FU. The parallel assessment of ESR2 mRNA and ERβ protein provided complementary information on transcriptional and protein-level responses, while the miRNA findings offered additional regulatory context.

The docking analysis provided exploratory structural context but does not demonstrate direct OL–ERβ binding, receptor activation, or a causal role of ERβ in the observed biological responses. Confirmation of the proposed relationship will require direct binding and receptor-specific functional studies, including ERβ knock-down and rescue experiments. The sample size was also insufficient for reliable multivariate modeling of ESR2 mRNA, ERβ protein, miRNAs, and tumor burden; these endpoints were therefore interpreted as complementary observations rather than evidence of causal or quantitatively correlated relationships. In addition, because the study was conducted exclusively in male BALB/c mice, possible sex-dependent differences remain to be established.

A repeated water-gavage control group was not included in the original design. Although distilled water served only as an inert administration medium, the effects of repeated handling and gavage-related stress cannot be fully distinguished from OL-specific effects when OL-treated groups are compared with untreated or single-injection 5-FU groups. These comparisons should therefore be interpreted as differences between the complete treatment regimens. The study was limited to predefined short-term endpoints and did not evaluate long-term survival or tumor recurrence; consequently, the OL pre-treatment findings should not be interpreted as evidence of a long-term preventive benefit. Finally, additional physiological, histopathological, oxidative-stress, and apoptosis outcomes were outside the analytical scope of the present report. The conclusions are therefore restricted to the tumor-related and molecular endpoints reported herein.

## 4. Conclusions

This study demonstrates that the biological activity of oleuropein in the Ehrlich ascites tumor model is strongly shaped by treatment timing and chemotherapy context. OL administered after tumor establishment increased viable tumor-cell burden, whereas 5-FU produced a marked antitumor response. Notably, concomitant OL administration substantially attenuated the 5-FU-associated reduction in viable tumor cells, indicating that OL does not act as a uniformly chemotherapy-synergistic phytochemical. In contrast, administration from tumor initiation or before tumor inoculation significantly reduced ascites accumulation, revealing distinct effects of OL on tumor-associated fluid dynamics depending on the treatment schedule.

At the molecular level, OL produced pronounced and temporally distinct regulation of the ESR2/ERβ pathway. Early OL exposure induced a coordinated increase in both ESR2 mRNA and ERβ protein, whereas prolonged prophylactic exposure produced transcriptional upregulation accompanied by reduced ERβ protein abundance, suggesting a transition toward additional post-transcriptional regulation. OL also reshaped miRNA responses: it markedly attenuated the strong 5-FU-induced increase in miR-146a-5p, while prolonged exposure significantly reduced miR-155-5p. The reciprocal pattern between miR-146a-5p and ERβ during 5-FU treatment identifies this miRNA as a particularly relevant candidate within the OL-responsive ERβ-associated regulatory network. The ensemble docking analysis showed that OL could be computationally accommodated within selected crystallographic ERβ ligand-binding-domain conformations. This observation provides a structural hypothesis for future investigation but does not demonstrate direct OL–ERβ binding or establish ERβ as the mediator of the observed biological responses. Direct binding assays, ERβ-dependent reporter experiments, ligand-competition studies and receptor loss-of-function approaches will be required to test this possibility. Together, the in vivo, molecular, and structural findings identify a treatment-dependent interplay among OL, ESR2/ERβ responses, miRNA regulation, and 5-FU response.

Overall, these results expand the understanding of oleuropein beyond a simple anticancer phytochemical and indicate that its biological consequences depend critically on when and in which therapeutic context it is administered. The study provides a mechanistic framework for further investigation of OL-responsive ERβ signaling and highlights treatment scheduling as an important consideration when evaluating oleuropein as an adjunct to conventional chemotherapy.

## Figures and Tables

**Table 1:** Effects of oleuropein and 5-FU on viable EAT-cell burden and ascites volume (n = 7 mice/group).

Group	Total Tumor Cell Count (×10^7^)	Ascites Fluid Volume (mL)
Untreated control	31.48 ± 5.83	4.58 ± 0.60
EAT + OL (6 days)	62.59 ± 6.70 *§	4.67 ± 0.56
EAT + 5FU (positive control)	3.96 ± 1.85 *	0.5 ± 0.13 *
EAT + 5FU + OL (6 days)	33.21 ± 5.21 §	2.84 ± 0.48 *
EAT + OL (8 days)	34.51 ± 5.65	2.40 ± 0.28 *
EAT + OL (15 days)	35.44 ± 3.84	2.63 ± 0.59 *

* *p* < 0.05 vs. untreated EAT control; § *p* < 0.05 vs. EAT + 5-FU.

**Table 2:** ESR2 mRNA and ERβ protein expression in EAT cells after oleuropein and/or 5-FU treatment.

Group	Relative mRNA Expression Level of Estrogen Receptor (FC: Fold Change)	Protein Expression Level (%) of Estrogen Receptor
Untreated control	1.04 ± 0.15	35.44 ± 0.95
EAT + OL (6 days)	1.41 ± 0.20	19.21 ± 0.08
EAT + 5FU	0.76 ± 0.15	11.57 ± 1.17 *
EAT + 5FU + OL (6 days)	1.55 ± 0.14 *§	32.11 ± 1.46 §
EAT + OL (8 days)	1.89 ± 0.13 *	73.88 ± 4.97 *
EAT + OL (15 days)	2.11 ± 0.37 *	27.38 ± 1.02 *

* *p* < 0.05 vs. untreated EAT control; § *p* < 0.05 vs. EAT + 5-FU.

**Table 3:** Relative miR-146a-5p and miR-155-5p expression in EAT cells after oleuropein and/or 5-FU treatment.

Groups	Relative Expression Value of miR-146a-5p (FC)	Relative Expression Value of miR-155-5p (FC)
Untreated control	1.00 ± 0.36	1 ± 0.19
EAT + OL (6 days)	0.93 ± 0.18 §	0.95 ± 0.21
EAT + 5FU	4.89 ± 0.27 *	0.72 ± 0.16
EAT + 5FU + OL (6 days)	1.97 ± 0.15 §	0.54 ± 0.31
EAT + OL (8 days)	1.43 ± 0.15	0.59 ± 0.06 *
EAT + OL (15 days)	1.47 ± 0.26	0.55 ± 0.14 *

* *p* < 0.05 vs. untreated EAT control; § *p* < 0.05 vs. EAT + 5-FU.

**Table 4:** Summary of the receptor structures, grid-center coordinates, redocking scores, and heavy-atom RMSD values used to evaluate the docking protocol.

PDB ID	Used Chain	Native Ligand	Grid Center (x,y,z)	Cavity Volume (Å3)	Binding Score (kcal/mol) *				Redocking Score (kcal/mol)	Heavy-Atom RMSD (Å)
					Oleuropein	Oleuropein Aglycone	Genistein	17β- Estradiol		
1QKM	A	Genistein	28, 11, 110	950	−9.3	−7.7	−9.6	−9.5	−9.9	0.242
3OLS	A	17β-Estradiol	26, −21, −15	1427	−6.9	−8.3	−9.2	−11.3	−11.4	0.411
5TOA	A	17β-Estradiol	17, 40, 11	946	−8.0	−8.0	−9.0	−11.1	−11.1	0.356

* More negative docking scores indicate more favorable predicted interactions within the applied scoring function. However, docking scores are computational estimates and do not represent experimentally measured binding affinities or direct evidence of ligand–receptor binding.

## Data Availability

The original contributions presented in this study are included in the article/Supplementary Materials. Further inquiries can be directed to the corresponding authors.

## Supplementary Materials

The following supporting information can be downloaded at https://www.mdpi.com/article/10.3390/biom16101420/s1, Figure S1: Representative CB-Dock2 binding poses of oleuropein, oleuropein aglycone, 17β-estradiol and genistein in the ERβ ligand-binding domains of 5TOA. Original Western blot images are also provided in the Supplementary Materials.
