# Supplementary material for: Oleuropein Modulates ESR2/ERβ-Associated microRNA Responses and Alters 5-Fluorouracil Activity in the Ehrlich Ascites Tumor Model: An In Vivo and Ensemble Docking Study
Source: Biomolecules. 2026 Oct 1;16(10):1420. doi: 10.3390/biom16101420 (PMC13702179; doi:10.3390/biom16101420)
Supplement: Supplementary file 1 [file biomolecules-16-01420-s001.zip › Figure S1.pdf]

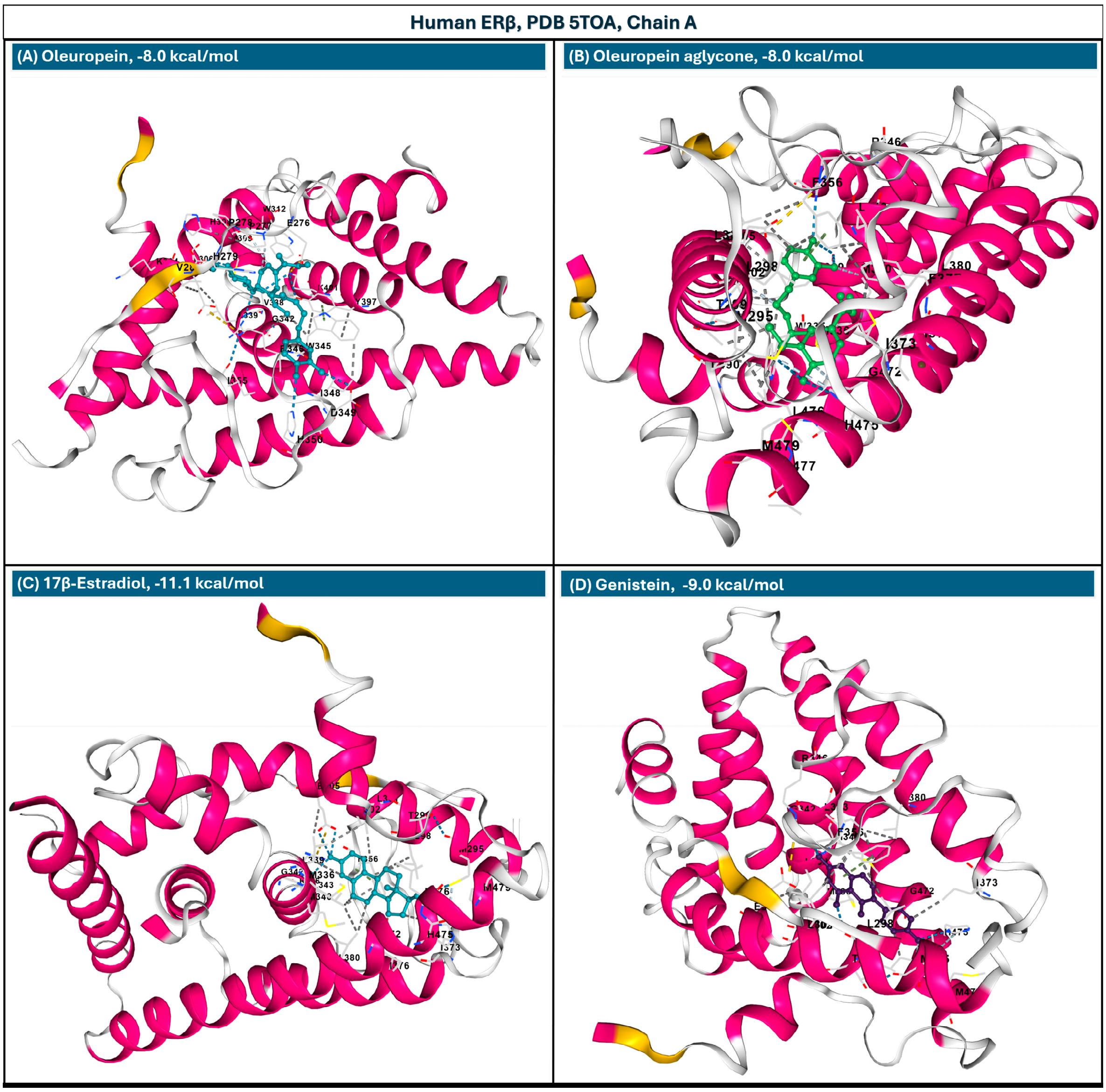

Figure S1. Representative CB-Dock2 binding poses of oleuropein, oleuropein aglycone, 17β-estradiol and genistein in the ERβ ligand-binding domains of 5TOA.
